# Trocar Site Hernia after Laparoscopic Colectomy: A Case Report and Literature Review

**DOI:** 10.5402/2011/725601

**Published:** 2011-05-29

**Authors:** Delmonaco Pamela, Cirocchi Roberto, La Mura Francesco, Morelli Umberto, Migliaccio Carla, Napolitano Vincenzo, Trastulli Stefano, Farinella Eriberto, Giuliani Daniele, Desol Angelo, Milani Diego, Di Patrizi Micol Sole, Spizzirri Alessandro, Bravetti Maurizio, Sciannameo Vito, Avenia Nicola, Sciannameo Francesco

**Affiliations:** General Surgery Department, St. Maria Hospital, University of Perugia, 05100 Terni, Italy

## Abstract

*Background*. Trocar Site Hernia (TSH) is defined as an incisional hernia which occurs after minimally invasive surgery on the trocar incision site.In 2004 Tonouchi classified trocar site hernias into 3 types: Early onset type; Late onset type; Special type. *Case Report*. We report the case of a 76-year old woman that underwent an emergency explorative laparotomy on the 10th p.o. day after a laparoscopic left hemicolectomy. Surgery showed a small bowel herniation through the 12 mm trocar incision site; the intestinal loop appeared necrotic and had to be resected, and the hernia orifice was repaired. We carried out a review of literature about this topic. *Discussion*. The clinical onset of a trocar site hernia is usually early, occurring within the 30th post operative day and it is caused by the omentum or small bowel entrapment into the trocar orifice. The clinical presentation is insidious, with progression to an acute abdomen, and an emergency surgical approach is often required. *Conclusions*. TSH is a severe complication of operative laparoscopy especially with large-bore trocar ports. The incidence of TSH resulting from our review ranges from 0.007% to 22% with an average of 1.85%. Prevention of TSH appears to be more effective when trocar insertion through the abdominal wall is tangential, the closure of both the fascia and the peritoneum is performed if the incision is greater than 7 mm, the suture of extra umbilical port site is performed under laparoscopic vision.

## 1. Background

Since laparoscopic surgery was introduced in 1987 by Mouret and more frequently employed, it has rapidly evolved as a major innovation in the history of surgery [1], offering an important contribution to the reduction of all the typical complications related to open surgery. On the other hand, laparoscopic surgery can be associated with a specific type of incisional hernia through the trocar site causing complications such as small bowel obstruction. Trocar site hernia (TSH) is defined as an incisional hernia which occurs after minimally invasive surgery on the trocar incision site [2]; some authors also define this condition as a port site hernia.

In 2004 Tonouchi classified trocar site hernias into 3 types [3] as follows.

**(i) Early onset type**

Dehiscence of anterior and posterior fascial plane and peritoneum characterized by early onset after surgery. It usually occurs as a small bowel obstruction. An example of this type is the so-called Richter's hernia.

**(ii) Late onset type**

Dehiscence of anterior and posterior fascial plane. Peritoneum constitutes the hernia sac. Hernias usually develop several months after surgery and they are not associated with small bowel obstruction. They appear as an asymptomatic swelling by the wound site.

**(iii) Special type**

Dehiscence of the whole abdominal wall. Intestine and/or omentum protrusion. There is no sac, being not a typical herniation. Its onset is very early, immediately after surgery.

## 2. Case Report

We report the case of a 76-year-old woman who underwent a laparoscopic left hemicolectomy for a sigmoid tumour. The patient was discharged in good general conditions and with open alvus on the 7th postoperative day.

**Figure 1:**
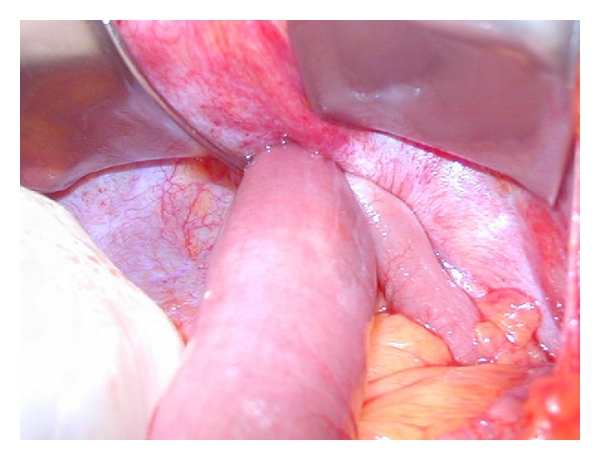


Three days after discharge, the patient was affected by colicky abdominal pain mostly localized in the left upper quadrant, vomiting, and constipation which required a rehospitalization. On the clinical examination the abdomen was distended and tender particularly in the left upper quadrant; an oval mass (5 cm) was detected in the same abdominal quadrant near the 12 mm trocar site. The ultrasound scan showed a swelling constituted by entrapped intestinal loops, and the abdominal X-ray revealed multiple small bowel gas-fluid levels. Because of the clinical, US and radiological signs of obstruction, the patient underwent an emergency explorative laparotomy which showed a small bowel herniation through the trocar incision site in the left upper quadrant (Figure 1); the intestinal loop appeared necrotic and had to be resected, and the hernia orifice was repaired. The patient was discharged on the 8th postoperative day.

## 3. Discussion

Trocar site hernia is a complication that is likely to be minimized [4]. 

The clinical onset of a trocar site hernia is usually early as it often occurs within the 30th postoperative day, and it is due to the omentum or small bowel entrapment into the trocar wound. The intestinal occlusion clinical presentation is often insidious, with progression to an acute abdomen, and an early and emergency surgical approach is often required [3–5].

The first trocar site hernia case was described by Fear in 1968 [6]. In this report, herniation of a small bowel loop was noticed in the immediate postoperative time. This was also the first case of the “special type hernia” described by Tonouchi. So this first report tended to express a protrusion of the bowel and/or omentum as a “hernia," although in the described type no hernia sac was detected [1–3]. 

In 1974 Schiff and Naftolin reported two cases of small bowel herniation occurred between the 14th and 21st postoperative days that required laparotomy with small bowel resection [7].

One of the first multicentric reports of operative laparoscopy revealed six incisional hernias out of 3560 cases (0.17%). These included four small bowel herniations and two omental herniations. Five of the six herniations were detected through a 12 mm port site, showing that the risk of herniation through a 12 mm trocar site was approximately 3-fold greater than that of a 10 mm trocar site. All the herniations were extraumbilical, and in three of the four cases of small bowel herniation, the fascia had been sutured. In this report Kadar et al. described cases of extraumbilical port site hernia, and for the first time two of the patients were treated laparoscopically [8].

In 1994 an amount of 933 hernias was reported from a 4,385,000 estimated laparoscopic procedures (0,02%) by the American Association of Gynecologic Laparoscopists. 167 (17.9%) were reported to have occurred despite fascial closure. 665 patients (71.3%) required a following surgical repair. 725 (86.3%) of the 840 hernias in which the size of the original fascial defect was noted occurred in sites where 10 mm or larger diameter trocars had been placed. Only 10.9% (92/840) were related to the use of 8–10 mm trocars and only 2.7% (23/840) to minor diameter trocars. The hernias occurrence in this report is a function of the number of the performed laparoscopic procedures (*P* < .0001), and it is not related to the surgeon's experience (*P* = .41) [9]. 

These data are shared by the study reported by Tonouchi; indeed, according to his work, 78.3% of hernias occurred on the 10–12 mm trocar site, while 21.7% occurred on the site of a trocar whose diameter was ≤5 mm [3]. 

In 1995, Boike et al. described 19 cases with 21 bowel herniation from 11 participating institutions. Two patients showed incisional herniation simultaneously on two port sites. Of the 21 herniations, 12 (57%) occurred on 12 mm port sites, 8 (38%) on 10 mm port sites, and one on 11 mm port site. Fascial screws were used to anchor ports in 11 (57%) patients with herniations. An attempt to close the fascia was performed in 9 patients (43%) during primary surgery. 16 herniations (76%) occurred on extraumbilical sites and 5 (23%) on the umbilical port site. The hernia contained small bowel in 18 cases (21%), cecum in 2 cases (0.9%), and ascending colon in 1 case (0.4%). The average interval to second operation was 8.5 days (range 2 to 42 days). In 3 patients (14%) the bowel herniation was repaired laparoscopically, while two patients (0,9%) required small bowel resection [4]. 

From 1995 till 1996, on a total of 32,205 gynaecological laparoscopies, 130 major complications were registered by the National Patient Insurance Association and 8 incisional hernias were reported (0.025%). The complications following operative laparoscopies included 48% urethral injuries, 19% bladder injuries, 13% intestinal injuries, 7% incisional hernias, 2% large-vessel injuries, and 11% different injuries. Incisional hernias were diagnosed between the 2nd and 14th postoperative days. The size of the trocars ranged from 5 to 12 mm; small bowel resection was needed in three patients (38%) [10].

Boughey et al. in 2003 described four cases of Richter's hernia after laparoscopy, two of which were repaired by open procedure and two by laparoscopy. The authors concluded that a laparoscopic hernia repair is an acceptable treatment at the time of diagnosis, especially in obese patients, as long as the incarcerated bowel is not severely compromised or ischemic [11].

In 2006, Immè and Cardi. studied 600 patients undergoing laparoscopic surgery. The incidence of incisional hernia was estimated around 2%, exclusively in the periumbilical area. No incisional hernia in extra-umbilical areas was registered, despite the authors did not use to perform fascial suture on extraumbilical sites. This study underlined the fact that particular attention needs to be paid to periumbilical gap suture which is exposed to the trauma of trocar fixing, especially in obese and diabetic patients. In selected cases the other gaps should be sutured, including the 5 mm ones [12].

Trocar site hernia is also one of the major complications after laparoscopic ventral hernia repair (LVHR). Its incidence was reported around 22% in a recent study published by Boldò et al. in 2007. This report showed a higher incidence in the TSH group of female gender, patients treated with large meshes, and patients affected by diabetes; but the use of meshes larger than 10 × 15 cm for LVHR was the only TSH risk factor to reach statistical significance. The cause for this finding is probably due to the dilatation of the trocar orifice during the introduction of the mesh and also to a postoperative mesh retraction [13].

Although trocar site hernias are well-known postoperative complications after laparoscopic surgery in adult patients, they have also been reported in preschool children. In 2008 Paya et al. conducted a retrospective reviewed of 293 laparoscopic procedures performed at a pediatric surgical tertiary care unit during a period of 4 years. 8 severe postoperative complications (2.7%) were described, and three of those were trocar site hernias (1.0%). All of them were omental hernias, and in all cases they were treated between the 3rd and 4th postoperative day. In all the patients, sharp-edged or pointedly round tips 2 to 5 mm sized trocars were used. Trocar site closure was performed using double-layer sutures for the first trocar, while the other trocar sites closure was performed carrying out one single suture including all layers [14].

Before this work there was only one published series, for children and adolescents up to 19 years of age, reporting an incidence of trocar site hernia of 0.3% (2 out of 574 patients) [15].

Although TSH in adult patients is mostly limited to ≥10 mm trocars, in 1999 Eltabbakh. reported the case of a 54-year-old woman who presented small bowel obstruction and herniation through a 5-mm trocar site 1 week after a laparoscopically assisted vaginal hysterectomy and bilateral salpingo-oophorectomy [16]. 

Since 1999 the purpose of several reports has been to focus on trocar-related problems with special respect to the tip design, concluding that port sites created by nonbladed trocars could not require fascial closure. Indeed, Kolata demonstrated that the wounds made by nonbladed trocars were narrower than those created by cutting tip trocars in a pig experimental model [17]. 

Leibl et al. compared two groups of patients treated in a nonrandomized design with either sharp cutting single-use trocars or cone-shaped noncutting reusable trocars. This trial showed an incisional hernia in 1.83% of patients treated with a sharp trocar tip, a complication which could be significantly lowered down to 0.17%, by using a conic tip design. So they demonstrated a reasonable benefit for a conic tip design, which enables an atraumatic insertion through the abdominal wall [18]. 

In 2000 Liu and McFadden reported 180 laparoscopic port sites performed with nonbladed trocars without fascial closure. Upon removal of large laparoscopic ports, the fascial defect was less than 6 to 8 mm, and the muscles of the abdominal wall covered the port site defect. The anterior fascial defect did not line up with the posterior fascial defect after removal of CO2 insufflation. No patient developed ventral incisional hernias in the postoperative period. The conclusion of the study was that the use of nonbladed laparoscopic trocars appears to be a safe technique, allowing to visualize dissection through the abdominal wall layers and to create the smallest port dissection without cutting muscle fibers and with no bleeding risk. The possibility to disrupt the abdominal wall musculature allows the surgeon to avoid the closure of the small fascial defect [19].

Johnson et al. performed a retrospective review of 747 operative procedures using VersaStep trocar system, one of nonbladed laparoscopic trocars, in patients undergoing Roux-en-Y gastric bypass surgery. There were no hernias detected at any of the 1494 12-mm or 2241 5-mm VersaStep trocar sites, despite lack of suture closure. At the Hasson port site, there was a hernia incidence of 1.20% [20].

In 2007 Shaher reviewed different wound closure techniques by a literature search [21]. In this review, old methods using classical instruments including Reverdin and Deschamps needles appeared to be cost effective and also useful as well as special wound devices designed for port site closure. Moreover, Elashry et al. and Nakada et al. showed in two randomized control studies that the Carter-Thomason device and the eXit puncture set both rated slightly higher than other devices; the Carter-Thomason device is also faster than all the tested techniques, results in lower port closure-related complications, and provides a leak-proof closure [21–23]. Insertion of a SURGICEL plug into the muscular layer of trocar wounds has also been proposed by Chiu et al. [24]. Alternatively, tangential insertion of a trocar through the abdominal wall might be effective in reducing the size of fascial defects [25, 26]. Moreover, different data from the literature have demonstrated that radially expanding type trocars could be useful to avoid the necessity of closing the fascial defect [27].

## 4. Conclusions

Trocar site hernia (TSH) is one of the potentially severe complications of operative laparoscopy with large-bore trocar ports. TSH has been infrequently reported in the setting of two-trocar procedures but appears to be more common in operative procedures with multiple large bore ports, and it may occur at both umbilical and extraumbilical sites (Table 3). 

The incidence of TSH is lower than incisional hernias after open surgery, even if the actual incidence could be probably higher. Indeed, our literature review points out at least 14 reports of TSH whose incidence is not reported (Table 1). Moreover, TSH was not listed or reported as a complication of laparoscopic surgery in several recent surveys. In addition, some patients, loss from followup is also to be considered, other patients may remain asymptomatic, while in other cases herniation might not be evident because of obesity. Besides all these underestimated cases, the incidence of TSH resulting from our review ranges from 0.007% to 22% (Table 1) with an average of 1.85%. 

The risk factors associated with the occurrence of TSH are related both to the patient's characteristics and the surgical technique. An important predisposing factor is obesity because of obese patients' thicker peritoneum, but advanced age, gender, nutritional status, diabetes, anaemia, steroid therapy, renal insufficiency, cancer, and wound infections also contribute to the occurrence of TSH. Different factors related to the surgical technique may also increase the risk of herniation by widening the fascial defect, such as the use of “fascial screws" to secure the port within the abdominal wall; longer procedures that result in excessive manipulation of port sites may also widen the fascial defect and increase the risk of herniation; large ports; cutting trocars; undetected omentum or bowel entrapment into the intraperitoneal defect after trocar removal; not sutured larger fascial defects (Table 2).

Omental herniation, a type 3 complication according to the classification of Tonouchi, may be an expected postoperative complication of laparoscopic surgery for young children. In adult surgery incisions ≤ 5 mm are sometimes not sutured at all, as in infants they are only sutured by one cutaneous stitch. According to most of the authors, despite the evidence that some types of trocars prevent or are better at avoiding trocar site herniation, closure of both the fascia and the peritoneum of the site should be, however, recommended if the incision is greater than 7 mm in adult patients and ≥5 mm in young children. 

Different authors believe that inserting the 10 mm lateral trocar in an oblique way or as a Z-tract will reduce hernia formation by placing the external and internal fascias at different levels; instead, according to recent reports, tangential insertion of a trocar through the abdominal wall might be effective in reducing the size of fascial defects making it easier to close the fascia and peritoneum at the same time. 

Unfortunately, although properly currently performed, closure is not always completely protective. Prevention of extraumbilical incisional hernias and dehiscences appears to be more effective when suture is performed under laparoscopic vision keeping the trocar inserted. Moreover, both the aponeurosis and the peritoneal membrane should be treated as carefully as possible, and different methods or devices should be employed to simplify and minimize the risk of hernia formation.

A careful postoperative management is recommended especially for patients with risk factors such as obesity, extensive manipulation of the trocar, and longer procedures. Bowel occlusion clinical presentation is often insidious, as in the case of a partial Richter's hernia. When in laparoscopic surgery a resolution of postoperative ileus after a period of 7–14 days of medical therapy and observation is not obtained, a differential diagnosis between postoperative ileus and pathologic postoperative occlusions (trocar site hernia, adhesions) is to be assessed. In these patients observation alone could be not enough appropriated to perform a well-timed diagnosis, making abdominal CT still play a key role for a right differential diagnosis.

##  Authors' Contributions

All authors contributed equally to this work.

## Figures and Tables

**Table 1:** Trocar site hernia frequency.

	Pub.	Patients undergoing VLS	Trocar site hernia/ Tot-VLS	TSH %
Fear	1968	Gynaecological surgery	1/NS	
Schiff and Naftolin	1974	Gynaecological surgery	2/NS	
Mintz (Review)	1977	Abdominal surgery	7/100000	0.007
Bourke	1977	Abdominal surgery	1/NS	
Sauer and Jarrett	1984	Diagnostic laparoscopy	1/NS	
Hogdall and Rosen	1987	Gynaecological surgery	1/NS	
Kiiholma, and Makinen	1988	Gynaecological surgery	1/NS	
Thomas et al.	1990	Abdominal surgery	1/NS	
Voyles	1991	Cholecystectomy	1/500	0.20
Larson	1992	Cholecystectomy	3/1983	0.15
Baird	1992	Cholecystectomy	1/800	0.13
Kadar et al.	1993	Gynaecological surgery	4/NS	
Multicentrico	1993	Gynaecological surgery	6/3560	0.17
Multicentrico	1994	Gynaecological surgery	933/4.385.000	0.021
George	1994	hysterectomy	3/NS	
Multicentrico	1995	Gynaecological surgery	19/NS	
Le Bouëdec	1995	Hysterectomy	1/NS	
Azurin	1995	Cholecystectomy	10/1300	0.77
Mike et al.	1996	Pediatric surgery	2/574	0.35
Mayol	1997	Abdominal surgery	6/403	1.48
Ahmad	1997	Cholecystectomy	11/1300	0.84
Nassar	1997	Cholecystectomy	16/870	1.83
Multicentric	1999	Gynaecological surgery	8/32.205	0.025
Sanz-Lopez	1999	Cholecystectomy	2/123	1.62
Berthou	1999	Colectomy for diverticulitis	1/110	0.90
Eltabbakh	1999	Gynaecological surgery	1/NS	
Coda	2000	Abdominal surgery	13/1287	1
Schauer	2000	Gastric bypass	1/257	0.36
Bowrey	2001	Fundoplication	9/320	2.8
Schmedt et al.	2001	Ventral hernia repair	301/6023	4.99
Berger et al.	2002	Incisional hernia repair	4/150	2.66
Al-Haijar	2002	Cholecystectomy	10/1453	0.68
Lumley	2002	Colectomy for neoplasm	1/152	0.65
Dresel	2002	Gastric banding	1/100	1
Susmallian,	2002	Gastric banding	3/459	0.65
Boughey et al	2003	Abdominal surgery	4/NS	
Immè, and Cardì F.	2006	Abdominal surgery	12/600	2
Boldó et al.	2007	Ventral hernia repair	6/27	22.2
ten Duis et al.	2008	Abdominal surgery	3/NS	
Paya et al.	2008	Pediatric surgery	8/293	2.73
Our experience	2009	Abdominal surgery	2/4387	0.045

VLS: laparoscopy

**Table 2:** Fascia closure.

Report	Cases	Fascia closure	Year
Kadar et al.	6	50%75%SBH	1993
George	3	100%	1994
Multicenter	933	17.9%	1994
Multicenter	19	42%	1995
Eltabbakh.	1 (5 mm)	0	1999
Immè and Cardì.	12	100%	2006
Our experience	2	0	2009

**Table 3:** TSH review.

Report	Cases	Port size	Site	PO day	Lap/VLS	SBR	Year
Schiff, Naftolin	2	NS	Umb	17.5	2/0	100%	1974
Bourke	1	12 mm	Umb	13	1/0		1977
Sauer, Jarret	1	NS	Umb	54	1/0		1984
Hogdall, and Rosen	1	12 mm	Umb	1	1/0	100%	1987
Kiiholma, and Makinen	1	10 mm	Umb	5	1/0		1988
Thomas et al.	1	NS	Umb	2	1/0	100%	1993
Kadar et al.	6	0.23% 10 mm 3.1% 12 mm	Ex-Umb	5	4/2		1993
Kurtz et al.	1	12 mm	Ex-Umb	6	1/0		1993
Montz	850	>10 mm (86.3%) 8–10 mm (10.9%) <8 mm (2.7%)					
George	3	12 mm	NS	2	0/3		1994
Multicentric	21*(19+ 2) *	12 mm (61%) 11 mm (4.8%) 10 mm (47,7%)	Umb (23.8) Ex-Umb (76.2%)	8.5	17/2	9.5%	1995
Le Bouëdec	1	12 mm	Ex-Umb	6	0/1		
Eltabbakh.	1	5 mm	Ex-Umb	7	1/0		1999
Multicentric	8	5 mm (12.5%) 10 mm (62.5%) 12 mm (25%)	Umb	8	8/0	38%	1999
Immè, and Cardi F	12	>10 mm	Umb	NS	12/0		2000
Our experience	2	12 mm	Ex-umb	12	2/0	50%	2009

LAP: laparotomy; VLS: laparoscopy.

SBR: small bowel resection.

Umb: Umbilical; Ex-Umb: extraumbilical.

## References

[1] Mouret P, Litynski GS (1996). Interview by GS Litynski. *Highlights in the History of Laparoscopy*.

[2] Crist DW, Gadacz TR (1993). Complications of laparoscopic surgery. *Surgical Clinics of North America*.

[3] Tonouchi H, Ohmori Y, Kobayashi M, Kusunoki M (2004). Trocar site hernia. *Archives of Surgery*.

[4] Boike GM, Miller CE, Spirtos NM (1995). Incisional bowel herniations after operative laparoscopy: a series of nineteen cases and review of the literature. *American Journal of Obstetrics and Gynecology*.

[5] Coda A, Bossotti M, Ferri F (2000). Incisional hernia and fascial defect following laparoscopic surgery. *Surgical Laparoscopy, Endoscopy and Percutaneous Techniques*.

[6] Fear RE (1968). Laparoscopy: a valuable aid in gynecologic diagnosis. *American Journal of Obstetrics and Gynecology*.

[7] Schiff I, Naftolin F (1974). Small bowel incarceration after uncomplicated laparoscopy. *Obstetrics and Gynecology*.

[8] Kadar N, Reich H, Liu CY, Manko GF, Gimpelson R (1993). Incisional hernias after major laparoscopic gynecologic procedures. *American Journal of Obstetrics and Gynecology*.

[9] Montz FJ, Holschneider CH, Munro MG (1994). Incisional hernia following laparoscopy: a survey of the american association of gynecologic laparoscopists. *Obstetrics and Gynecology*.

[10] Härkki-Siren P, Sjöberg J, Kurki T (1999). Major complications of laparoscopy: a follow-up Finnish study. *Obstetrics and Gynecology*.

[11] Boughey JC, Nottingham JM, Walls AC (2003). Richter’s hernia in the laparoscopic era: four case reports and review of the literature. *Surgical Laparoscopy, Endoscopy and Percutaneous Techniques*.

[12] Immè A, Cardì F (2006). Incisional hernia at the trocar site in laparoscopic surgery. *Chirurgia Italiana*.

[13] Boldó E, Perez de Lucia G, Aracil JP (2007). Trocar site hernia after laparoscopic ventral hernia repair. *Surgical Endoscopy*.

[14] Paya K, Wurm J, Fakhari M, Felder-Puig R, Puig S (2008). Trocar-site hernia as a typical postoperative complication of minimally invasive surgery among preschool children. *Surgical Endoscopy*.

[15] Chen MK, Schropp KP, Lobe TE (1996). Complications of minimal-access surgery in children. *Journal of Pediatric Surgery*.

[16] Eltabbakh GH (1999). Small bowel obstruction secondary to herniation through a 5-mm laparoscopic trocar site following laparoscopic lymphadenectomy. *European Journal of Gynaecological Oncology*.

[17] Kolata RJ, Ransick M, Briggs L, Baum D (1999). Comparison of wounds created by non-bladed trocars and pyramidal tip trocars in the pig. *Journal of Laparoendoscopic and Advanced Surgical Techniques—Part A*.

[18] Leibl BJ, Schmedt CG, Schwarz J, Kraft K, Bittner R (1999). Laparoscopic surgery complications associated with trocar tip design: review of literature and own results. *Journal of Laparoendoscopic and Advanced Surgical Techniques—Part A*.

[19] Liu CD, McFadden DW (2000). Laparoscopic port sites do not require fascial closure when nonbladed trocars are used. *The American Surgeon*.

[20] Johnson WH, Fecher AM, McMahon RL, Grant JP, Pryor AD (2006). VersaStep*™* trocar hernia rate in unclosed fascial defects in bariatric patients. *Surgical Endoscopy*.

[21] Shaher Z (2007). Port closure techniques. *Surgical Endoscopy*.

[22] Elashry OM, Nakada SY, Wolf JS, Figenshau RS, McDougall EM, Dayman RV (1996). Comparative clinical study of port-closure techniques following laparoscopic surgery. *Journal of the American College of Surgeons*.

[23] Nakada SY, McDougall EM, Gardner SM, Gonzalez G, Clayman RV (1995). Comparison of newer laparoscopic port closure techniques in the porcine model. *Journal of Endourology*.

[24] Chiu CC, Lee WJ, Wang W, Wei PL, Huang MT (2006). Prevention of trocar-wound hernia in laparoscopic bariatric operations. *Obesity Surgery*.

[25] Bowrey DJ, Blom D, Crookes PF (2001). Risk factors and the prevalence of trocar site herniation after laparoscopic fundoplication. *Surgical Endoscopy*.

[26] Hogdall C, Roosen JU (1987). Incarcerated hernia following laparoscopy. *Acta Obstetricia et Gynecologica Scandinavica*.

[27] Johnson WH, Fecher AM, McMahon RL, Grant JP, Pryor AD (2006). VersaStep*™* trocar hernia rate in unclosed fascial defects in bariatric patients. *Surgical Endoscopy*.

